# Impacts of Outsourcing Medication Repackaging in Nursing Homes: Quality and Areas of Pharmacy–Nursing Collaboration

**DOI:** 10.3390/pharmacy13060182

**Published:** 2025-12-13

**Authors:** Thomas Schmid, Falk Hoffmann, Michael Dörks, Kathrin Jobski

**Affiliations:** 1 Faculty of Social and Health Studies, University of Applied Sciences Kempten, 87435 Kempten, Germany; thomas.schmid@hs-kempten.de; 2 Department of Health Services Research, Faculty VI Medicine and Health Sciences, Carl von Ossietzky University Oldenburg, 26129 Oldenburg, Germany; falk.hoffmann@uni-oldenburg.de (F.H.); michael.doerks@uni-oldenburg.de (M.D.)

**Keywords:** medication repackaging, medication organizer, dose dispensing, interprofessional collaboration, nursing, pharmaceutical staff, nursing homes

## Abstract

The task of repackaging resident’s medication into medication organizers is increasingly outsourced from nursing homes to pharmacies, presenting an opportunity to redefine the interaction between nursing and pharmaceutical staff. This study investigated whether outsourcing medication repackaging changes the quality and subjects of collaboration between the two professions. A cross-sectional survey was developed targeting heads of nursing in German nursing homes. A simple random sample of 1415 nursing homes was contacted by phone. Respondents participated either by phone or by online survey. Quality of collaboration was measured using Kenaszchuk’s Interprofessional Collaboration Scale (ICS) with its subscales Communication, Accommodation and Isolation. Topics of interaction were ascertained using items along a medication management phase model. Differences in response frequencies were analyzed using Fisher’s exact test. A total of 268 nursing homes participated (response: 18.9%). Of these, 132 (49.3%) had outsourced repackaging. Respondents at nursing homes with in-house medication repackaging rated the subscale Accommodation more favorably (*p* = 0.008), while Communication and Isolation showed no difference. Of the 13 individual ICS items, “passing on information” (Communication) was rated better by respondents at homes with outsourced repackaging (*p* = 0.019) and “consideration of convenience” (Accommodation) more favorably by respondents at homes with in-house repackaging (*p* = 0.042). Nursing staff at homes with outsourced medication repackaging interacted with pharmaceutical staff more frequently on medication changes (*p* < 0.001), but less frequently on tablet splitting (*p* = 0.035). In conclusion, outsourcing medication repackaging has a limited impact on the quality of interprofessional collaboration between the two professions but may have the potential to reduce ambiguities regarding splitting tablets.

## 1. Introduction

Collaboration among healthcare professionals has the potential to reduce errors and improve care [1,2]. Among other determinants, organizational factors are considered drivers of the quality of interprofessional collaboration in healthcare [3,4]. In accordance with previous developments in the Nordic countries and the Netherlands [5,6,7], the medication supply process in German nursing homes has recently undergone major organizational changes. While previously it was the nursing homes that repackaged residents’ medication from the original packs provided by community pharmacies into medication organizers, the repackaging process is increasingly outsourced to community pharmacies, which in turn often resort to industrial, (semi-) automated dose-dispensing providers to perform the task. Recent research has found that 51.5% of German nursing homes have outsourced the repackaging of medication into medication organization devices to community pharmacies [8].

This transition is likely to have altered the organizational arrangements of both the affected nursing homes and the supplying pharmacies, e.g., with respect to the number of pharmacies that supply medication to an individual nursing home, the responsibilities of the involved healthcare providers and operating procedures, implying potential effects on the quality of collaboration between the professions involved [2,3,4,9].

In addition to organizational changes and their potential effects on the quality of interprofessional interaction, the trend toward outsourcing medication repackaging in German nursing homes may also have had an impact on the topics of interaction between the two professions. The German health technology assessment agency Institute for Quality and Efficiency in Health Care has suggested that the repackaging of medication by pharmacies might be associated with improved medication therapy in some studies, because it is inextricably linked to improved medication management [10]. Thus, outsourcing medication repackaging could have, e.g., raised the priority of medication risks in the interaction between nursing and pharmaceutical staff.

Given this background, this study aimed at investigating whether the quality and topics of collaboration between nursing homes’ nursing staff and pharmaceutical staff at the supplying community pharmacies differ depending on whether nursing homes in- or outsource the repackaging of their resident’s medication.

## 2. Materials and Methods

### 2.1. Design and Recruitment

The study was designed as a cross-sectional survey of German nursing homes. In February 2023, 11,088 nursing homes offering permanent long-term care were retrieved from the nursing home registry of Germany’s largest statutory health insurance provider AOK. A simple random sample of nursing homes was created using a random number generator. Starting with the lowest number, nursing homes were contacted by phone and invited to participate. The interviewers asked to be put through to the head of nursing or their deputy as eligible participants. If the first contact by phone failed, a second attempt was made. Upon obtaining informed consent, the survey was conducted either during the phone call by the interviewer or by the head or deputy head of nursing after the phone call using an online survey, if the participant preferred this option. In the latter case, the respondent used the same online entry mask as the interviewers.

### 2.2. Survey Development and Content

A survey was developed that included 45 items on quality and topics of interaction between nursing and pharmaceutical staff, as well as nursing homes’ characteristics. Filling was expected to take approximately 15 min.

The quality of the relationship between the nursing homes’ nursing staff and the pharmaceutical staff at community pharmacies was recorded using the Interprofessional Collaboration Scale [11] in its German version [12]. This constitutes a validated survey instrument [13] which was designed for use with multiple groups of health professionals [14], including nurses as informants on the quality of collaboration with pharmaceutical staff [11]. The survey consists of 13 items that can be aggregated into the subscales Communication, Accommodation, and Isolation [11,13]. Response options to the items are “strongly disagree”, “disagree”, “agree” and “strongly agree” [11]. The survey items, corresponding subscales and short names used to refer to the items in the subsequent passages are outlined in Table 1. Responses to the item “The pharmaceutical staff think their work is more important than the work of our nursing staff” were reverse-coded in the analysis, since it was the only item phrased negatively in the German version [12].

Survey items on the topics of collaboration between nursing staff and pharmaceutical staff were developed along a medication process model with the stages prescription, dispensing, medication administration and monitoring/review, building on literature on medication repackaging for nursing homes’ residents [15,16,17,18]. These stages were complemented by invoicing as an item category. In the survey section on interaction topics, participants were asked to assess how frequently their nursing staff interacted with pharmaceutical staff on a five-point scale, ranging from “very rarely” to “very frequently”. In addition, a response option “no opinion/don’t know” was offered to participants to express uncertainty about an item. The survey items related to interaction topics are listed in Table 2.

To characterize the structural and organizational attributes of the participants’ nursing homes, additional survey items covered responsibility for repackaging and managing follow-on prescriptions, number of collaborating pharmacies, number of appointments with collaborating pharmacies per year, predominant type of medication organization device in use, number of different medications per resident, ownership, community size, federal state and the home’s number of care recipients.

The survey was pre-tested with two heads of nursing and a director of a nursing home to ensure the comprehensibility of the survey items and validate the duration of the survey. The survey did not require significant modifications based on the results of these pre-tests.

### 2.3. Statistical Analysis

Descriptive statistics (percentages, median and interquartile range (IQR)) were used to characterize the respondents’ nursing homes. Characteristics of facilities that repackaged their residents’ medication themselves were compared with those of nursing homes that outsourced the repackaging. *p*-values were ascertained using *t*-test (continuous variables) and Fisher’s exact test (dichotomous and categorical variables). Statistical significance was determined at a *p*-value of less than 0.05.

Responses to the subscales of the Interprofessional Collaboration Scale were analyzed using descriptive statistics (response frequencies, category percentages). Differences in responses by participants at nursing homes with in- versus outsourced medication repackaging were assessed using Fisher’s exact test. The differentiated analysis of the subscales of the Interprofessional Collaboration Scale was stratified by number of care recipients and restricted to reusable rigid medication organizers to check for facility size and medication organizer type as mediating factors. Individual items of the Interprofessional collaboration scale and the items on topics of interaction were also examined using descriptive statistics (response frequencies, category percentages) and Fisher’s exact test, again differentiating the facilities according to in- versus outsourced medication repackaging. For items on topics of interaction, answers indicating uncertainty were excluded, i.e., if a respondent chose the option “no opinion/don’t know”, the response was not considered in the statistical analysis.

All analyses were carried out using SAS for Windows Version 9.4 (SAS Institute Inc., Cary, NC, USA).

## 3. Results

### 3.1. Respondent Characteristics

A simple random sample of 1415 nursing homes were contacted by phone between March 2023 and January 2024 and invited to participate. In total, 268 heads or deputy heads of nursing responded to the survey (response: 18.9%). Of these, 197 (73.5%) participated by phone and 71 (26.5%) used the online form to fill in the survey; 40 (14.9%) respondents stated that their nursing home is located in a community with a population of more than 100,000, while 83 (31.0%), 96 (35.8%) and 49 (18.3%) worked for nursing homes in communities with a population of 20,000–99,999, 5000–19,999 and less than 5000, respectively (Table 3). Some 87 (32.5%) of the respondents’ nursing homes operated under private ownership, 146 (54.5%) under non-profit and 35 (13.1%) under public ownership. The respondents’ nursing homes served a mean of 74.2 care recipients, each taking a median of 6 different long-term medications.

While 115 (42.9%) used mainly reusable rigid medication organizers, 69 (25.7%) used pouches/sachets, 41 (15.3%) unsealed dosage cups and 28 (10.4%) blister cards as medication organizers. In terms of responsibilities, 156 (58.2%) answered that their nursing homes were primarily responsible for managing follow-on prescriptions, whereas 112 (41.8%) respondents stated that this fell to a pharmacy. While 214 (79.9%) responded that their home worked with only one pharmacy as their medication supplier, 37 (13.8%) stated the number of supplying pharmacies as two, and 17 (6.3%) stated the number of supplying pharmacies as three or more. In addition, the median number of formal appointments per year between nursing homes and supplying pharmacies amounted to two.

### 3.2. Characteristics of Nursing Homes with In-House Versus Outsourced Medication Repackaging

While 136 (50.7%) stated that their nursing home’s staff repackages residents’ medication into medication organizers, 132 (49.3%) answered that theirs had outsourced the repackaging to a pharmacy.

The two groups differed primarily in size of facility, the medication organizers used, the responsibility for obtaining follow-on prescriptions, as well as the number of supplying pharmacies. Homes with insourced medication repackaging had a mean number of residents of 68.4, while those with outsourced medication repackaging had 80.3 (*p* = 0.006). Homes that repackaged the medication themselves used primarily reusable rigid medication organizers (68.4%) such as dosettes, whereas the predominant type of medication organizers in homes with outsourced repackaging was pouches (also known as sachets), at 52.3% (*p* < 0.001).

In the majority of cases, the institutional responsibility for obtaining follow-on prescriptions coincided with that for medication repackaging. When nursing homes repackaged medications into organizers themselves, 92.6% of the facilities also took care of follow-on prescriptions as compared to 22.7% when this was outsourced (*p* < 0.001). Among the respondents’ nursing homes repackaging medication themselves, 70.6% worked together with only one pharmacy, while 89.4% of nursing homes with outsourced repackaging were supplied by only one pharmacy (*p* < 0.001).

Additional nursing home characteristics referring to quality assurance and pharmacies’ compensation for repackaging are outlined in Tables S1 and S2.

### 3.3. Quality of Collaboration

When respondents were not differentiated by responsibility for repackaging, the perceived quality of collaboration between nursing staff and pharmaceutical staff was generally high (Table S3). When discerning the two groups, the perceived quality of collaboration differed only marginally between respondents at nursing homes with in-house medication repackaging and those with outsourced repackaging. While the subscales Communication and Isolation showed no difference in the frequencies of response categories between the two groups, the ratings on the subscale Accommodation differed in that respondents at homes with outsourced medication repackaging more frequently expressed strong disagreement with the survey items (*p* = 0.008, Table 4). When the analysis of the quality subscales was further broken down into large versus small nursing homes or restricted to reusable rigid medication organizers, no differences could be found between facilities with in-house versus outsourced medication repackaging (Table S4).

**Figure 1:**
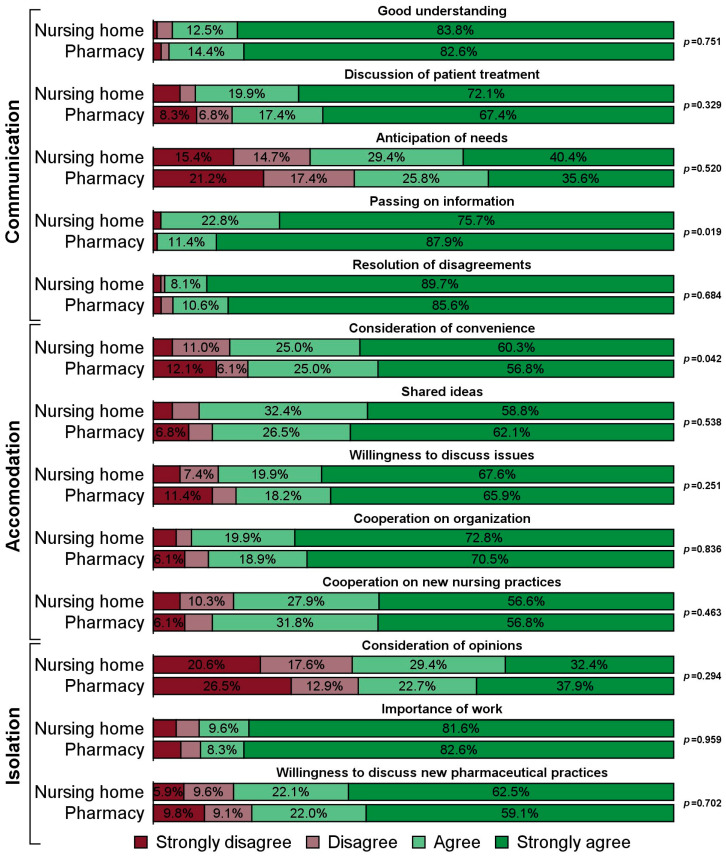
Response frequencies to individual items regarding the quality of collaboration, according to responsibility for repackaging residents’ medication (nursing home with N = 136 versus pharmacy with N = 132).

With respect to the 13 individual survey items forming the subscales, respondents at nursing homes with in-house medication repackaging gave different answers than those with outsourced repackaging for the two items “passing on information” (Communication) and “consideration of convenience” (Accommodation). Specifically, the group of respondents with outsourced medication repackaging more frequently strongly agreed to the statement that important information was always passed on between their nursing staff and the pharmaceutical staff (*p* = 0.019). Conversely, respondents at nursing homes with in-house medication repackaging more often strongly agreed that the pharmaceutical staff were usually willing to take into account the convenience of their institution’s nursing staff when planning their work (*p* = 0.042, Figure 1).

### 3.4. Topics of Collaboration

**Figure 2:**
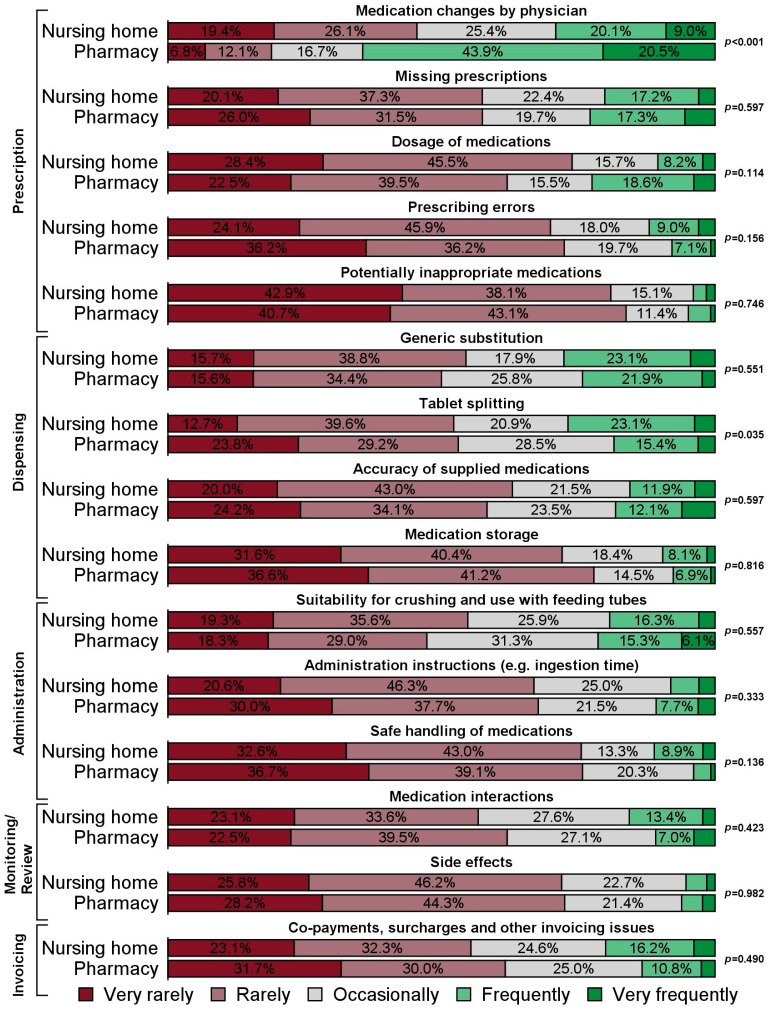
Response frequencies to items regarding the topics of interaction, according to responsibility for repackaging residents’ medication (nursing home versus pharmacy). Note: Analyses are based on certain answers only (i.e., respondents choosing the option “no opinion/don’t know” were excluded from the respective analyses). The number of certain answers for the individual items are displayed in Table S5.

The subjects of interaction receiving the highest percentage of “very frequent” or “frequent” responses were medication changes (46.7%), generic substitution (25.9%) and tablet splitting (22.7%, Table S5). Only minor differences, yet related to two of the most common subjects of interaction, existed between facilities with in-house and outsourced medication repackaging. First, fewer respondents with insourced medication repackaging indicated that they interact “frequently” or “very frequently” with pharmaceutical staff on medication changes by physicians than those at homes with outsourced medication repackaging (29.1% vs. 64.4%, *p* < 0.001). Second, a larger percentage of respondents with in-house medication repackaging reported being in contact with pharmaceutical staff “frequently” or “very frequently” regarding tablet splitting (26.8% vs. 18.5%, *p* = 0.035, Figure 2).

## 4. Discussion

### 4.1. Key Findings

This study found that 49.3% of German nursing homes have outsourced repackaging their residents’ medication from original packs into medication organizers to community pharmacies. Those nursing homes have also commonly outsourced the management of follow-on prescriptions, used markedly different medication organizers and worked with fewer supplying pharmacies.

The quality of collaboration between the care homes’ nursing staff and the pharmaceutical staff at the supplying pharmacies was generally high and differed only marginally between nursing homes with in-house and those with outsourced medication repackaging. The subscale Accommodation and the corresponding item “consideration of convenience” were rated more favorably by nursing homes with in-house medication repackaging, while the item “passing on information” pertaining to the subscale “Communication” was rated better by respondents at nursing homes with outsourced medication repackaging.

In care homes that outsourced medication repackaging, contacts regarding medication changes were indicated as being more frequent, whereas contacts about tablet splitting were more frequent in homes repackaging medication in-house.

Attributes of nursing homes with in-house versus outsourced medication repackaging.

The percentage of German nursing homes having outsourced repackaging their residents’ medication to community pharmacists matches previous research [8]. However, the dissemination of outsourcing medication repackaging is still well below that of the Netherlands where the percentage of nursing homes that outsource medication repackaging has recently been determined as 72.3% [8]. Moreover, our results are also in line with other research that has found the predominant use of disposable medication organizers such as pouches when nursing facilities outsource repackaging to pharmacies [19]. The differences between nursing homes with in-house and outsourced medication repackaging in the type of medication organizers used, the management of follow-on prescriptions and the number of supplying pharmacies support the initial premise of this study that increased outsourcing of medication repackaging has led to major organizational changes in those nursing homes’ medication supply process.

### 4.2. Quality of Collaboration

There were no suitable comparison studies that have investigated nurses’ ratings of pharmaceutical staff on the scale used in this research. However, the respondents’ perceived quality of collaboration with pharmaceutical staff was quite high compared to other studies using the Interprofessional Collaboration Scale and investigating nurses as informants on allied health professions [14,20].

The organizational changes resulting from outsourcing medication repackaging have presented an opportunity to standardize and formalize processes as well as roles and responsibilities between the two professions involved, which in turn could have enhanced interprofessional collaboration [2,3,4,9]. The marginal differences in perceived quality of collaboration between nursing homes with in-house versus outsourced medication repackaging indicate that these potential improvements have not materialized, or improvements have been outweighed by other factors not immediately related to interprofessional collaboration. Even more so, as the stratified analyses of large versus small homes and the restriction to rigid reusable medication organizers suggest that facility size and medication organizer type act as mediators for the observed differences in the Accommodation subscale.

Thus, the results stand in contrast to the effectiveness of other organizational interventions with an explicit intention of improving interprofessional collaboration, such as interprofessional training or interprofessional medication reviews involving the two health professions [21,22].

Having found few differences in the quality of collaboration between nursing staff and pharmaceutical staff depending on the responsibility for repackaging medication, our results take a middle ground between three studies from the Nordic countries. In a survey conducted in Norway after the introduction of an automated multi-dose drug dispensing system operated by external suppliers, 84% of nursing staff found that routines had improved [15]. In line with these results, another Norwegian study surveying nurses after the implementation of a multidose drug dispensing system with an external repackaging facility inferred that the change in the system had created responsibility and new uniform collaboration routines [16]. In contrast to the results from Norway, a study in Sweden found that one third of the nurses working with multidose medication organizers supplied by pharmacies agreed that the opportunity to communicate with pharmacies could be improved in this setup [17].

The respondents’ ratings in our study on the item “passing on information” (Communication) and the subscale Accommodation could be explained by organizational and technological necessities rather than conscious redefinitions of processes, roles and responsibilities when nursing homes outsource repackaging to pharmacies. When nursing home wards keep little or no medication stock anymore due to the outsourcing of repackaging, the institutions’ nursing staff inevitably have to interact more frequently with pharmacies when medications are changed. Along the same lines, if repackaging medication is outsourced, the nursing staff is inevitably less involved in the medication process [18], making it more difficult to accommodate their desire to be knowledgeable about residents’ medication [16,19]. Likewise, if automated filling of medication organizers by pharmacies necessitates the use of pouches or other disposable medication organizers, the preferences of those nursing staff favoring reusable rigid organizers, such as dosettes, cannot be met. However, additional research is required to test these hypotheses.

### 4.3. Topics of Interaction

Frequent interaction about medication changes between nursing staff and the pharmaceutical staff responsible for repackaging medication is in line with past research that has found such changes a challenge when the repackaging process is outsourced [16,17]. This can be explained by lower or no ward stocks due to outsourced repackaging.

Less frequent contact between nursing homes with outsourced medication repackaging and pharmacies regarding tablet splitting is a more remarkable result of this study. Tablet splitting is a widespread practice [23,24,25] which is often executed inappropriately and can result in serious hazards for patients and caregivers [26,27]. Nurses commonly feel that they lack the information resources to adequately modify solid dosage forms [28]. They often refer patients to a pharmacist, especially when it comes to sustained release or coated tablets which are more likely not to be suitable for splitting [29]. Hence, the lower contact frequency found in this study between nurses and pharmaceutical staff when nursing homes have outsourced medication repackaging can be interpreted as a reduced need to consult with pharmacies on tablet splitting, since pharmaceutical staff independently decide which tablets can be split before repackaging them into medication organizers. Moreover, the results suggest that nurses that repackage medications themselves could benefit from additional training on tablet splitting and better access to specific medication information.

### 4.4. Overall Assessment of the Relationship Between Nursing and Pharmaceutical Staff

Both the topics and quality section of this study suggest a rather technical nature of the current relationship between nursing staff at nursing homes and pharmaceutical staff at supplying pharmacies. From the perspective of topics, there are few subjects apart from medication changes with very frequent or frequent interaction between the two groups of health professionals. Considering the quality of collaboration, the results of this study on the impacts of outsourcing medication repackaging on the Accommodation scale can be interpreted as technical and organizational inevitabilities. From a theoretical perspective, this raises the question whether in the nursing home setting the relationship between nursing staff and pharmaceutical staff can be described as interprofessional collaboration, at all, or whether it should rather be considered a case of interprofessional coordination.

### 4.5. Strengths and Limitations

As a major strength, this study is the first to provide comprehensive insights on the impacts of outsourcing medication repackaging in nursing homes on quality and topics of interprofessional collaboration between nursing and pharmaceutical staff using a random sample. A key limitation of this study lies in the survey instrument used to measure quality of collaboration, given it was developed in a hospital setting and tested considering allied health professionals as a homogeneous group [11]. Moreover, the response was at 18.9%, which was likely attributable to persistent nursing staff shortages in German nursing homes as well as heavy workload of eligible respondents, thus limiting the generalizability of the results. In addition, the views of the pharmaceutical staff were not included in the study. Furthermore, the range “very rarely” to “very frequently” used for the topics of interaction items does not allow for conclusions on absolute frequencies of interaction (e.g., once per month). Finally, the study is subject to the restrictions of a cross-sectional perspective. To corroborate the results, an additional longitudinal approach investigating nursing homes before and after outsourcing medication repackaging would be advisable.

## 5. Conclusions

Nursing homes with outsourced medication repackaging evaluated the quality of collaboration with pharmaceutical staff slightly less favorably than those with in-house repackaging. Nursing staff in nursing homes with outsourced medication repackaging interacted more frequently with pharmaceutical staff on medication changes, but less frequently on tablet splitting than in facilities with in-house medication repackaging. In conclusion, the nursing homes’ tendency to outsource medication repackaging has little impact on the quality of interprofessional collaboration between the facilities’ nursing staff and the pharmaceutical staff at supplying pharmacies, but has the potential to reduce ambiguities with respect to splitting tablets.

## Figures and Tables

**Table 1:** Survey items on collaboration, subscales and item short names.

Subscale/Item Text	Item Short Name
**Communication**	
Our nursing staff have a good understanding with the pharmaceutical staff about our respective responsibilities.	Good understanding
I feel that patient treatment and care are not adequately discussed between our nursing staff and the pharmaceutical staff. *	Discussion of patient treatment
The pharmaceutical staff anticipate when our nursing staff will need their help.	Anticipation of needs
Important information is always passed on between our nursing staff and the pharmaceutical staff.	Passing on information
Disagreements with the pharmaceutical staff often remain unresolved. *	Resolution of disagreements
**Accommodation**	
The pharmaceutical staff are usually willing to take into account the convenience of our nursing staff when planning their work.	Consideration of convenience
Our nursing staff and the pharmaceutical staff share similar ideas about how to treat patients.	Shared ideas
The pharmaceutical staff are willing to discuss our nursing staff’s issues.	Willingness to discuss issues
The pharmaceutical staff cooperate with the way we organize our nursing staff’s care.	Cooperation on organization
The pharmaceutical staff would be willing to cooperate with new nursing practices.	Cooperation on new nursing practices
**Isolation**	
The pharmaceutical staff do not usually ask for our nursing staff’s opinions. *	Consideration of opinions
The pharmaceutical staff think their work is more important than the work of our nursing staff. ^+^	Importance of work
The pharmaceutical staff would not be willing to discuss their new practices with our nursing staff. *	Willingness to discuss new pharmaceutical practices

* Phrased negatively in English, but positively in German, + phrased negatively in both English and German, reverse coded.

**Table 2:** Survey items on interaction topics grouped by medication phase.

How Often is the Nursing Staff in Contact with the Supplying Pharmacy/Pharmacies on the Following Topics?
**Prescription** Medication changes by physician Missing prescriptions Dosage of medications Prescribing errors Potentially inappropriate medications
**Dispensing** Generic substitution Tablet splitting Accuracy of supplied medications Medication storage
**Administration** Suitability for crushing and use with feeding tubes Administration instructions (e.g., ingestion time) Safe handling of medications
**Monitoring/review** Medication interactions Side effects
**Invoicing** Co-payments, surcharges and other invoicing issues

**Table 3:** Nursing home characteristics.

	Medication Repackaged by			
	Nursing Home	Pharmacy		Overall
	**N = 136**	**N = 132**	*p*-Value	**N = 268**
Community size			0.608	
≥100,000	19 (14.0%)	21 (15.9%)		40 (14.9%)
20,000–99,999	38 (27.9%)	45 (34.1%)		83 (31.0%)
5000–19,999	53 (39.0%)	43 (32.6%)		96 (35.8%)
<5000	26 (19.1%)	23 (17.4%)		49 (18.3%)
Type of ownership			0.718	
Private	44 (32.4%)	43 (32.6%)		87 (32.5%)
Non-profit	72 (52.9%)	74 (56.1%)		146 (54.5%)
Public	20 (14.7%)	15 (11.4%)		35 (13.1%)
Number of care recipients				
Mean (Std)	68.4 (33.5)	80.3 (36.7)	**0.006**	74.2 (35.6)
Median (IQR)	66 (44–88)	78 (54–101)		71 (50–93)
Number of different medications per resident				
Mean (Std)	6.2 (1.6)	5.7 (1.9)	**0.012**	5.9 (1.8)
Median number of different medications per resident (IQR)	6 (5–7)	6 (5–7)		6 (5–7)
Medication organizer types			**<0.001**	
Reusable rigid medication organizer	93 (68.4%)	22 (16.7%)		115 (42.9%)
Pouch/sachet	0 (0.0%)	69 (52.3%)		69 (25.7%)
Unsealed dosage cup	36 (26.5%)	5 (3.8%)		41 (15.3%)
Blister card	0 (0.0%)	28 (21.2%)		28 (10.4%)
Other	7 (5.1%)	8 (6.1%)		15 (5.6%)
Management of follow-on prescriptions by			**<0.001**	
Nursing home	126 (92.6%)	30 (22.7%)		156 (58.2%)
Pharmacy/pharmacies	10 (7.4%)	102 (77.3%)		112 (41.8%)
Number of supplying pharmacies			**<0.001**	
1	96 (70.6%)	118 (89.4%)		214 (79.9%)
2	26 (19.1%)	11 (8.3%)		37 (13.8%)
3+	14 (10.3%)	3 (2.3%)		17 (6.3%)
Number of appointments with supplying pharmacies per year				
Mean (Std)	2.0 (2.0)	2.1 (2.0)	0.667	2.1 (2.0)
Median (IQR)	2 (1–2)	2 (1–2)		2 (1–2)

Bold font indicates *p*-values < 0.05.

**Table 4:** Response frequencies to subscales on the quality of collaboration, according to responsibility for repackaging residents’ medication (nursing home versus pharmacy).

Subscale	Response				*p*-Value
	Strongly disagree	Disagree	Agree	Strongly agree	
Communication (5 items)					0.214
Nursing home (N = 136)	4.9%	4.3%	18.5%	72.4%	
Pharmacy (N = 132)	6.7%	5.6%	15.9%	71.8%	
Accommodation (5 items)					**0.008**
Nursing home (N = 136)	4.4%	7.4%	25.0%	63.2%	
Pharmacy (N = 132)	8.5%	5.0%	24.1%	62.4%	
Isolation (3 items)					0.294
Nursing home (N = 136)	10.3%	10.5%	20.3%	58.8%	
Pharmacy (N = 132)	13.9%	8.6%	17.7%	59.9%	

Bold font indicates *p*-values < 0.05.

## Data Availability

The raw data supporting the conclusions of this article will be made available by the authors on request.

## Supplementary Materials

The following supporting information can be downloaded at: https://www.mdpi.com/article/10.3390/pharmacy13060182/s1, Table S1: Structural and organizational attributes when medication is repackaged by a pharmacy; Table S2. Structural and organizational attributes when medication is repackaged in the nursing home; Table S3. Response frequencies to individual items regarding the quality of collaboration (N=268); Table S4: Response frequencies to subscales on the quality of collaboration, according to responsibility for repackaging residents’ medication (nursing home versus pharmacy) (a) stratified by nursing home size and (b) restricted to nursing homes using reusable rigid medication organizers; Table S5. Response frequencies to items regarding the topics of interaction.
