# Supplementary material for: Impacts of Outsourcing Medication Repackaging in Nursing Homes: Quality and Areas of Pharmacy–Nursing Collaboration
Source: Pharmacy (Basel). 2025 Dec 13;13(6):182. doi: 10.3390/pharmacy13060182 (PMC12737295; doi:10.3390/pharmacy13060182)
Supplement: Supplementary file 1 [file pharmacy-13-00182-s001.zip › pharmacy-3981035-supplementary.pdf]

pharmacy

MDPI

Supplementary Materials:

Table S1. Structural and organizational attributes when medication is repackaged by a pharmacy.

	**N = 132**
Repackaging site	
In rooms at the care facility	6 (4.5 %)
At the pharmacy/ elsewhere	126 (95.5 %)
Checking of medication repackaged by pharmacy	
Yes	130 (98.5 %)
No	2 (1.5 %)
Time to check organizers filled by pharmacy	**N = 130**
Median in minutes (Q1-Q3)	6 (5-15)
Compensation for pharmacy	**N = 132**
Pharmacy receives service fee	52 (39.4 %)
No compensation	11 (8.3 %)
No information on compensation	69 (52.3 %)
Compensation for pharmacy	**N = 42**
Median (Q1-Q3)	€1.60 (€1.00-€3.00)

Table S2. Structural and organizational attributes when medication is repackaged in the nursing home.

	**N = 136**
The correct filling of MODs is usually checked by	
The same nurse	49 (36.0 %)
Another nurse	87 (64.0 %)
Median time for repackaging in minutes (Q1-Q3)	12 (10-20)

Table S3. Response frequencies to individual items regarding the quality of collaboration (N=268).

**Subscale / item short name**	**Response**			
	Strongly disagree	Disagree	Agree	Strongly agree
Communication	5.8 %	4.9 %	17.2 %	72.1 %
Good understanding	1.1 %	2.2 %	13.4 %	83.2 %
Discussion of patient treatment	6.7 %	4.9 %	18.7 %	69.8 %
Anticipation of needs	18.3 %	16.0 %	27.6 %	38.1 %
Passing on information	1.1 %	0.0 %	17.2 %	81.7 %
Resolution of disagreements	1.5 %	1.5 %	9.3 %	87.7 %
Accommodation	6.4 %	6.2 %	24.6 %	62.8 %
Consideration of convenience	7.8 %	8.6 %	25.0 %	58.6 %
Shared ideas	5.2 %	4.9 %	29.5 %	60.5 %
Willingness to discuss issues	8.2 %	6.0 %	19.0 %	66.8 %
Cooperation on organization	5.2 %	3.7 %	19.4 %	71.6 %
Cooperation on new nursing practices	5.6 %	7.8 %	29.9 %	56.7 %
Isolation	12.1 %	9.6 %	19.0 %	59.3 %
Consideration of opinions	23.5 %	15.3 %	26.1 %	35.1 %
Importance of work	4.9 %	4.1 %	9.0 %	82.1 %
Willingness to discuss new pharmaceutical practices	7.8 %	9.3 %	22.0 %	60.8 %

Table S4. Response frequencies to subscales on the quality of collaboration, according to responsibility for repackaging residents' medication (nursing home versus pharmacy) (a) stratified by nursing home size and (b) restricted to nursing homes using reusable rigid medication organizers.

**Subscale**	**Response**				**p-Value**
	**Strongly disagree**	**Disagree**	Agree	Strongly agree	
Smaller nursing homes*					
Communication (5 items)					0.430
Nursing home (N=74)	4.6 %	3.0 %	18.7 %	73.8 %	
Pharmacy (N=58)	6.2 %	4.5 %	15.5 %	73.8 %	
Accommodation (5 items)					0.232
Nursing home (N=74)	4.1 %	6.5 %	23.5 %	66.0 %	
Pharmacy (N=58)	7.2 %	4.5 %	22.1 %	66.2 %	
Isolation (3 items)					0.795
Nursing home (N=74)	9.9 %	9.9 %	20.3 %	59.9 %	
Pharmacy (N=58)	11.5 %	7.5 %	19.0 %	62.1 %	
Larger nursing homes					
Communication (5 items)					0.684
Nursing home (N=62)	5.2 %	5.8 %	18.4 %	70.7 %	
Pharmacy (N=74)	7.0 %	6.5 %	16.2 %	70.3 %	
Accommodation (5 items)					0.064
Nursing home (N=62)	4.8 %	8.4 %	26.8 %	60.0 %	
Pharmacy (N=74)	9.5 %	5.4 %	25.7 %	59.5 %	
Isolation (3 items)					0.394
Nursing home (N=62)	10.8 %	11.3 %	20.4 %	57.5 %	
Pharmacy (N=74)	15.8	9.5 %	16.7 %	58.1 %	
Use of reusable rigid medication organizers					
Communication (5 items)					0.338
Nursing home (N=93)	5.6 %	4.1 %	17.0 %	73.3 %	
Pharmacy (N=22)	1.8 %	5.5 %	15.5 %	77.3 %	
Accommodation (5 items)					0.235
Nursing home (N=93)	4.1 %	6.2 %	23.4 %	66.2 %	
Pharmacy (N=22)	1.8 %	3.6 %	18.2 %	76.4 %	
Isolation (3 items)					0.410
Nursing home (N=93)	10.8 %	9.3 %	20.8 %	59.1 %	
Pharmacy (N=22)	7.6 %	6.1 %	15.2 %	71.2 %	

* nursing homes where the number of care recipients is lower than 71, bold font indicates p-values <0.05

Table S5. Response frequencies to items regarding the topics of interaction.

**Item**	**Response**				
	Very rarely	Rarely	Occasionally	Frequently	Very frequently
Prescription					
Medication changes by physician (N = 266)	13.2 %	19.2 %	21.1 %	32.0 %	14.7 %
Missing prescriptions (N = 261)	23.0 %	34.5 %	21.1 %	17.2 %	4.2 %
Dosage of medications (N = 263)	25.5 %	42.6 %	15.6 %	13.3 %	3.0 %
Prescribing errors (N = 260)	30.0 %	41.2 %	18.9 %	8.1 %	1.9 %
Potentially inappropriate medications (N = 249)	41.8 %	40.6 %	13.3 %	3.2 %	1.2 %
Dispensing					

Item	Response				
	Very rarely	Rarel y	Occa-sionally	Fre-quently	Very fre-quently
Generic substitution (N = 262)	15.7 %	36.6 %	21.8 %	22.5 %	3.4 %
Tablet splitting (N = 264)	18.2 %	34.5 %	24.6 %	19.3 %	3.4 %
Accuracy of supplied medications (N = 267)	22.1 %	38.6 %	22.5 %	12.0 %	4.9 %
Medication storage (N = 267)	34.1 %	40.8 %	16.5 %	7.5 %	1.1 %
Administration					
Suitability for crushing and use with feed-ing tubes (N = 266)	18.8 %	32.3 %	28.6 %	15.8 %	4.5 %
Administration instructions (e.g. ingestion time) (N = 266)	25.2 %	42.1 %	23.3 %	6.4 %	3.0 %
Safe handling of medications (N = 263)	34.6 %	41.1 %	16.7 %	6.1 %	1.5 %
Monitoring / review					
Medication interactions (N = 263)	22.8 %	36.5 %	27.4 %	10.3 %	3.0 %
Side effects (N = 263)	27.0 %	45.3 %	22.1 %	3.8 %	1.9 %
Invoicing					
Co-payments, surcharges and other invoic-ing issues (N = 250)	27.2 %	31.2 %	24.8 %	13.6 %	3.2 %

Ns refer to the number of certain answers (i.e. respondents choosing the option “no opinion/don’t know” were excluded from the analyses)
